# Investigations of Personality Trait in Subacute Post-Stroke Patients: Some Preliminary Observations

**DOI:** 10.3390/medicina58050683

**Published:** 2022-05-20

**Authors:** Viviana Lo Buono, Francesca Noto, Lilla Bonanno, Caterina Formica, Francesco Corallo

**Affiliations:** IRCCS Centro Neurolesi Bonino Pulejo, 98100 Messina, Italy; francynoto@hotmail.it (F.N.); lilla.bonanno@irccsme.it (L.B.); caterina.formica@irccsme.it (C.F.); francesco.corallo@irccsme.it (F.C.)

**Keywords:** stroke, behavioral disorders, personality, health outcomes

## Abstract

Background and Objectives: Personality change is an important psychiatric complication following stroke linked to severe affective dysregulation and behavioral alterations. Methods: We investigated personality traits in 20 patients (age 45.37 ± 13.41 years) with subacute stroke submitted to rehabilitation training within 1–3 months after a first-onset stroke. All patients underwent psychological evaluation by using the Personality Inventory for DSM-5 for adults (PID-5), a specific instrument that enables traits (dimensions and facets) to be assessed by providing a personality profile, and the Inventory of Interpersonal Problems 47 (IIP-47), a brief and valid self-report measure for screening personality disorders. Results: Personality change was identified by a positive correlation IIP-47 and PID-5 (r = 0.76; *p* = 0.03). Our patients, after a stroke, presented maladaptive personality traits associated with negative affect such as anxiety, emotional lability, and rigid perfectionism, and they reported interpersonal problems. These negative affective disorders correlated positively with cluster C personality disorders, including the avoidant, dependent, and obsessive compulsive personality disorders. Conclusion: Preliminary results show personality changes in stroke survivors. The evaluation of personality changes could be useful to improve the management of the patient’s behavioral alterations in a familiar environment and permit the possibility of prevention of psychological distress of the patients and their respective caregivers.

## 1. Introduction

Personality is defined as sets of behaviors, cognitions, and emotional patterns specific to the individual [1]. Emotions and behavior can change after an acquired brain injury. Therefore, the concept of personality change following stroke refers to an alteration or discontinuity in personhood post cerebral lesion [2].

Personality change due to brain injury is an important psychiatric complication linked to severe affective dysregulation that could be due to the direct physiological effects of the brain damage [3,4]. Neuropsychiatric sequelae, including behavioral impairments such as poor motivation, apathy, and a tendency to be self-centered and less aware of the needs of others, are also commonly observed in patients following a stroke [5]. Patients, after stroke, can become disinhibited, agitated, or aggressive, and anxiety and depressive symptoms are quite frequent [6]. Some literature data support the idea that brain injury could be a risk factor for psychotic disorders [7,8,9].

The location of the brain lesion plays an important role in personality change. Lesions in the orbitofrontal cortex (OFC) areas are associated with important changes in behavior, such as increased impulsivity and a lack of judgment [10]. Neuroimaging studies suggest that the OFC may show a role in the modulation of behaviors related to clinical symptoms, such as increased aggression, anxiety, and depression after brain damage [11,12,13]. 

About two-thirds of stroke survivors show motor or cognitive disability [14]. Studies on personality change after stroke are lacking, although it may be expected that self-concept will change as individuals experience a differing relationship with their bodies in relation to disability.

We present preliminary data from an ongoing study on personality traits in subacute stroke patients submitted to neurocognitive rehabilitation training. 

## 2. Materials and Methods

This preliminary study included twenty patients (10 ischemic and 10 hemorrhagic, 42% female, age 45.37 ± 13.41 years, education 11.75 ± 3.53) admitted to a rehabilitation program within 1–3 months after a first-onset stroke. Exclusion criteria included severe aphasia, agnosia, neglect, and psychiatric history of cognitive impairment (MOCA score ≥ 20). 

All information related to brain lesions was collected by means of interviews and the examination of medical records.

Each patient underwent psychological evaluation for analysis of personality using: the Personality Inventory for DSM-5 for adults (PID-5) (Krueger et al., 2012) and the Inventory of Interpersonal Problems 47 (IIP-47), [15].

The PID-5-Adult is a 220-item self-rated personality trait assessment scale for adults aged 18 and older. It assesses 25 personality trait facets, including: anhedonia, anxiousness, attention seeking, callousness, deceitfulness, depressitivity, distractibility, eccentricity, emotional lability, grandiosity, hostility, impulsivity, intimacy avoidance, irresponsibility, manipulativeness, perceptual dysregulation, perseveration, restricted affectivity, rigid perfectionism, risk-taking, separation insecurity, submissiveness, suspiciousness, unusual beliefs and experiences, and withdrawal. Certain triplets of facets (groups of 3) can be combined to assess the five domains of traits: negative affect, detachment, antagonism, disinhibition and psychoticism [16]. 

The IIP-47 is a self-report measure for screening personality disorders through assessing interpersonal sensibility, interpersonal ambivalence, aggression, need for social approval, and lack of sociability [17]. 

### Statistical Analysis

Continuous variables were expressed as mean ± standard deviation. A nonparametric analysis was carried out because the results of the Shapiro–Wilk test normality test indicate that most of the target variables were not normally distributed. Correlations between variables were computed by Spearman’s coefficient between the sub-item of IIP-47 and the sub-item of PID-5. Analyses were performed using an open source R3.0 software package. A 95% confidence interval level was set with a 5% alpha error. Statistical significance was set at *p* < 0.05.

## 3. Results

**Figure 1:**
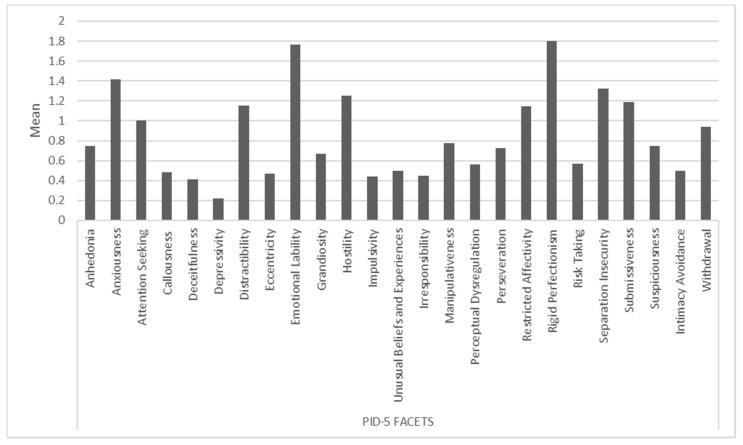
Representation of distribution of sub-items of PID-5 facets.

**Figure 2:**
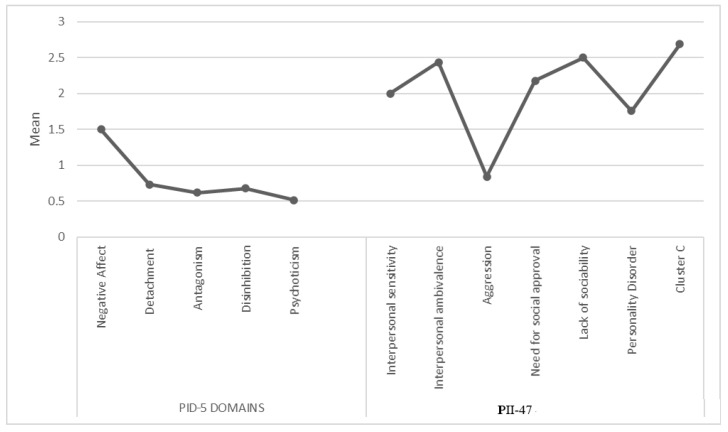
Representation of distribution of sub-items of PID-5 domains and sub-items of IIP-47.

The mean distribution of sub-items of PID-5 facets is shown in Figure 1, while Figure 2 shows sub-items of the PID-5 domains and IIP-47. Spearman correlation highlighted a significant positive correlation between sub-items of IIP-47 and sub-items of PID-5 domains, in particular between cluster C and negative affect (r = 0.76; *p* = 0.03).

## 4. Discussion

This preliminary study describes the changes in personality characteristics, such as alterations in how the person thinks, feels, and behaves, in stroke patients. Usually, an external stimulus brings about this modification in self-perception and may occur with violent tendencies, confusion, depression and apathy, psychotic symptoms, and relational closure.

Personality changes have been widely described after traumatic brain injury [18], since the way we process and understand information or process our emotions can change as a result of brain damage. Our patients, after a stroke, presented maladaptive personality traits associated with negative affect such as anxiety, emotional lability and rigid perfectionism and they reported interpersonal problems. These negative affective disorders correlated positively with cluster C personality disorders, including the avoidant, dependent, and obsessive compulsive personality disorders [19]. Negative affectivity refers to the tendency to experience negative emotions or use dysfunctional coping. Among them, emotional lability, anhedonia, distractibility and anxiety constitute vulnerability and maintenance factors in well-being following stroke, implying that personality may play a role in mental physical comorbidity.

Similarly, [20] reported negative behaviors in stroke patients, including sadness, disinhibition, lack of adaptation, environmental withdrawal, crying, passivity and aggressiveness. Maladaptive personality traits are most important for understanding health problems, as they described clinically significant personality characteristics. Indeed, maladaptive traits, such as neuroticism, may impede recovery from brain lesions, while protective traits such as extraversion and conscientiousness can facilitate the rehabilitative process [21]. 

Stroke causes long-term negative health outcomes, including physical disabilities and functional impairment and increased risk of mental disorders [9].

In addition, numerous stroke survivors present with emotional deficit, personality changes or inadequate behaviors. These disorders can change over time or be improved by medication. Sometimes, they permanently alter the personality or behavior of the person. The management of mood and behavior disorders is difficult for both stroke survivors and their caregivers. At the same time, these neuropsychiatric complications are often unaddressed or not adequately treated because of the preoccupation with physical disabilities, whereas treating these behavioral alterations after stroke can improve the overall outcome of the patients to a considerable extent.

Personality traits represent prospective predictors of health outcomes in the general population [22]. However, the current understanding of associations between personality and health is largely unexplored. 

A multidimensional approach taking into account neurological, psychological, and social perspectives could permit an understanding of psychopathological symptoms following stroke and increase the development of evidence-based therapeutic strategies to improve clinical outcomes and therapeutic management. 

The study has some limitations, including the small sample size, which do not allow the generalization of the results obtained. In addition, it would be necessary to carry out a longitudinal study to verify the long-term effects of stroke on personality dimensions. 

## 5. Conclusions

Personality can be well-defined as a dynamic set of self-representations, which are formed through personal experiences, and interpretation of the environment. After a brain injury, certain life stories are interrupted, the sense of coherence is undermined, and the future becomes uncertain and unpredictable [14]. 

The evaluation of personality changes could be useful to improve the management of the patient’s behavioral alterations in a familiar environment and permit the possibility of preventing psychological distress of the patients and their respective caregivers.

## Data Availability

All data generated or analyzed during this study are included in this published article.
